# Gut microbiota contributes to bisphenol A-induced maternal intestinal and placental apoptosis, oxidative stress, and fetal growth restriction in pregnant ewe model by regulating gut-placental axis

**DOI:** 10.1186/s40168-024-01749-5

**Published:** 2024-02-17

**Authors:** Hao Zhang, Xia Zha, Bei Zhang, Yi Zheng, Mabrouk Elsabagh, Hongrong Wang, Mengzhi Wang

**Affiliations:** 1 Laboratory of Metabolic Manipulation of Herbivorous Animal Nutrition, College of Animal Science and Technology, Yangzhou University, Yangzhou, 225009 P. R. China; 2 Joint International Research Laboratory of Agriculture and Agri-Product Safety, the Ministry of Education of China, Yangzhou University, Yangzhou, 225009 P. R. China; 3 Department of Animal Production and Technology, Faculty of Agricultural Sciences and Technologies, Niğde Ömer Halisdemir University, Nigde, 51240 Turkey; 4 Department of Nutrition and Clinical Nutrition, Faculty of Veterinary Medicine, Kafrelsheikh University, KafrelSheikh, Egypt; 5 State Key Laboratory of Sheep Genetic Improvement and Healthy Production, Xinjiang Academy of Agricultural Reclamation Science, Shihezi, 832000 P. R. China

**Keywords:** Bisphenol A, Fetal growth restriction, Gut microbiota, Gut-placental axis, Pregnant ewe

## Abstract

**Background:**

Bisphenol A (BPA) is an environmental contaminant with endocrine-disrupting properties that induce fetal growth restriction (FGR). Previous studies on pregnant ewes revealed that BPA exposure causes placental apoptosis and oxidative stress (OS) and decreases placental efficiency, consequently leading to FGR. Nonetheless, the response of gut microbiota to BPA exposure and its role in aggravating BPA-mediated apoptosis, autophagy, mitochondrial dysfunction, endoplasmic reticulum stress (ERS), and OS of the maternal placenta and intestine are unclear in an ovine model of gestation.

**Results:**

Two pregnant ewe groups (*n* = 8/group) were given either a subcutaneous (sc) injection of corn oil (CON group) or BPA (5 mg/kg/day) dissolved in corn oil (BPA group) once daily, from day 40 to day 110 of gestation. The maternal colonic digesta and the ileum and placental tissue samples were collected to measure the biomarkers of autophagy, apoptosis, mitochondrial dysfunction, ERS, and OS. To investigate the link between gut microbiota and the BPA-induced FGR in pregnant ewes, gut microbiota transplantation (GMT) was conducted in two pregnant mice groups (*n* = 10/group) from day 0 to day 18 of gestation after removing their intestinal microbiota by antibiotics. The results indicated that BPA aggravates apoptosis, ERS and autophagy, mitochondrial function injury of the placenta and ileum, and gut microbiota dysbiosis in pregnant ewes. GMT indicated that BPA-induced ERS, autophagy, and apoptosis in the ileum and placenta are attributed to gut microbiota dysbiosis resulting from BPA exposure.

**Conclusions:**

Our findings indicate the underlying role of gut microbiota dysbiosis and gut-placental axis behind the BPA-mediated maternal intestinal and placental apoptosis, OS, and FGR. The findings further provide novel insights into modulating the balance of gut microbiota through medication or probiotics, functioning via the gut-placental axis, to alleviate gut-derived placental impairment or FGR.

Video Abstract

**Supplementary Information:**

The online version contains supplementary material available at 10.1186/s40168-024-01749-5.

## Introduction

Developmental insults, stemming from malnutrition, stress, disease states, and environmental factors, may result in adverse birth outcomes including fetal growth restriction (FGR), preterm birth, and low birth weight. Moreover, these insults increase risk factors associated with disease onset in adulthood, according to the disease and heath hypothesis. Bisphenol A (BPA; 4,4-dihydroxy-2,2-diphenylpropane), an environmental pollutant with endocrine-disrupting properties, is widely used in numerous consumer products, including polyvinyl chloride, plastics, water bottles, food packaging, toys, medical devices, thermal receipts, and dental sealants. Human beings are subjected to BPA exposure via diverse routes, like dermal absorption, diet, or household dust inhalation [1]. Exposure to BPA has been shown to hamper mammary gland growth, metabolism, reproduction, and cognition [2]. Also, BPA may penetrate the placenta, adversely affecting its growth and function, leading to FGR, preeclampsia, or recurrent abortion [3]. Furthermore, gestational BPA exposure has been associated with unfavourable birth outcomes in various animal and human cohort studies [4–7]. Intrinsically, placentation in primates differs from that in ruminants [8]. However, sheep have been recognized as the ideal models for studying placental function due to the presence of multinucleate cells within their placental trophoblast layer, resembling the syncytiotrophoblast placental layer in humans [9].

The placenta, a transient organ anchoring the fetus to the reproductive tract wall, has a multifaceted role in facilitating the exchange of gas, nutrients, and waste [10]. Impairments of some placental function mediators may hinder placental development or essential processes necessary for meeting the metabolic needs of the growing fetus, causing FGR along with low birth weight in the offspring [11]. As reported in one in vivo model, the BPA-fed pregnant mice (dosage, 50 mg kg^−1^ BW) had reduced placental thickness [12]. Gestational exposure to BPA at the environmentally relevant dose up-regulates the negative placental function mediators and destroys placental steroidogenesis and angiogenesis, thus reducing the sheep’s placental efficiency [13].

BPA is mostly recognized as a strong endocrine disruptor [14], nonetheless, its dietary exposure may induce alterations in mouse gut microbiome, and the elevated Bacteroides abundances are related to metabolic diseases in the host [15]. A low Firmicutes/Bacteroidetes ratio was found to be associated with cardiovascular health, a balanced immune system, younger age, and lean phenotypes and is generally considered beneficial for health [16]. Gut microbiota affects gut immunity, inflammation, oxidative stress (OS), and intestinal barrier function by modulating the host-microbe interactions [17]. Gut microbiota imbalance or dysbiosis is associated with chronic inflammatory disorders, metabolic syndrome, or even complicated pregnancy [18]. The sudden alterations in the gut microbiome may induce preeclampsia, incidental damage to placental function, and preterm birth [19]. Based on the above context, the gut microbiome has a critical role in embryo and fetus development and is tightly associated with successful pregnancy. Accordingly, our study hypothesized that the “gut-placenta” axis is an important window for visualizing the FGR etiology. Yet, how BPA alters the gut microflora of pregnant ewes and the possible consequences of this alteration on maternal gut and placental function and fetal development remained unclear, which will be investigated in our current study.

Our previous research indicated that gestational BPA exposure in sheep induces autophagy, apoptosis, mitochondrial dysfunction, endoplasmic reticulum stress (ERS), and OS of placenta and trophoblasts and causes FGR [7]. These deleterious effects of BPA may be partially attributed to its impact on maternal gut microbiota. Consequently, our current work focused on exploring the roles of the gut microbiome and gut-placental axis in BPA-mediated maternal placental apoptosis, OS, and FGR in the pregnant ewe model. Further, we adopted the gut microbiota transplantation (GMT) technique [20] where gut microbiota from pregnant ewes exposed to BPA were orally administrated into pregnant mice to highlight the relationship between the gut microbiota and placental function.

## Materials and methods

Every experiment was performed in line with guidelines from the Institutional Animal Care and Use Committee of Yangzhou University (Ethical approval number: SYXK 2016–0019).

### Animals

The Hu ewes were raised within the facility of the Jiangyan Experimental Station of Taizhou, Jiangsu Province of China where the experiments were conducted. The housing facility was equipped with indoor heating radiators to maintain the temperature at 15 ± 0.5 ◦C throughout the study period while the illumination was under automatic adjustment to simulate the natural photoperiod [21]. Thirty-two multiparous Hu ewes of similar age (18.7 ± 0.6 months), body weight (BW 40.6 ± 1.8 kg), and body condition score (BCS 2.52 ± 0.15; scales 0–5 represent emaciation to obesity, respectively; [22]) were used. Ewes were dewormed using ivermectin (0.2 mg/kg BW) and synchronized for estrus using intravaginal progestagen sponges (30 mg; Pharmp PTY, Herston City, Australia). Sponges were removed after 12 days and ewes were exposed to teaser rams for monitoring the estrous behavior at 08:00 and 16:00 h over 24 h after sponge removal. At 48 h post-sponge removal, ewes were artificially inseminated with freshly collected semen and then placed into individual pens (1.2 m × 1.8 m) until day 40 of gestation on which the fetus number carried by every ewe was counted by ultrasonography (Asonics Microimager 1000 sector scanning instrument; Ausonics Pty Ltd., Sydney, Australia). There were 16 ewes carrying twin lambs. Every ewe was fed according to NRC’s nutrient recommendations for pregnant ewes [23] during days 0–40 of gestation. Ewes were fed at 08:00 once daily and allowed to drink clean water. Table S1 displays the experimental diet composition. More detailed information regarding feeding can be obtained elsewhere [24].

### Experimental design for pregnant ewes

On day 40 of gestation, twin-bearing ewes were randomized into 2 treatment groups of 8 ewes each and received either corn oil (Control, CON group) or BPA (purity ≥ 99%, Aldrich Chemical, Milwaukee, WI, USA) dissolved in corn oil at 5 mg/kg/day (BPA group). The daily dose was injected subcutaneously and the treatments continued from day 40 to day 110 of gestation. The BPA exposure time and dose are adopted from previous studies on pregnant ewes [7, 21].

### Sample collection from pregnant ewes

Ewes were stunned with a captive-bolt gun (Supercash Mark 2; Acceles and Shelvoke Ltd., Sutton Coldfield, England) before exsanguination on day 110 of gestation. Ewes has a cotyledonary placenta which is comprised of multiple small areas of contact between maternal (Caruncle) and fetal (Cotyledon) circulation systems forming button-like structures called placentomes. These placentomers are classified into types A, B, C, and D [25]. Cotyledons (COT) samples were collected from multiple type A placentomes having the same size within 10 cm of umbilical attachment, frozen in liquid nitrogen, and kept under – 80 ℃ according to a previous description [7, 26]. The maternal duodenal, jejunal, ileal, and colonic tissues and contents were collected, rapidly frozen in liquid nitrogen, and preserved under − 80 ℃. In the morning just before exsanguination (on day 110 of gestation), blood samples were collected from the jugular vein of each ewe into anticoagulant-free, sterile vacuum tubes (Vacutainer; Becton, Dickinson and Company, Suzhou, China) using the 20 gauge × 3.8-cm blood collection needles (Vacutainer; Becton, Dickinson and Company). Plasma was separated, through centrifugation of blood samples for 15 min at 3000 × *g* and 4 °C, and preserved under – 80 °C until analysis. The fetal body weight was determined and fetal sex distribution was analyzed in both treatment groups.

### Gut microbiota transplantation (GMT)

Pregnant ewes from both treatment groups were used as fecal donors for GMT to pregnant mice. In brief, approximately 100 mg digesta were collected from the CON and BPA ewes’ colons and resuspended with 1.5 mL sterile anaerobic PBS and 10-min centrifugation at 3000 × *g* and 4 ℃ to obtain microbial supernatants. These supernatants were filtered through a 70-μm filter [19, 27] followed by a counting test on a blood cell counting plate to ensure that the concentration of microbes in the suspension was more than 10^8^ CFU/mL. Finally, the microbial suspensions from the CON and BPA ewes were mixed separately, 10% glycerin was added, and the samples were stored at – 80 °C for conducting the GMT to mice. The GMT experiment was conducted on 7-week-old CD-1 mice (Beijing Vital River) in a specific pathogen-free environment under the conditions of 20 °C ± 3 °C, humidity of 55% ± 5% and light/dark cycle of 12-h/12-h following a previous method after modification [28]. In brief, the 7-week-old female mice were given intragastric administration of antibiotics (200 μL) once a day for consecutive 5 days (vancomycin, 100 mg/kg; neomycin sulfate, ampicillin, and metronidazole, 200 mg/kg). Thereafter, qPCR was conducted to detect bacterial total 16S rRNA for verifying the complete removal of enteric microbes following antibiotic treatment [29]. Fecal supernatants were orally inoculated into recipient mice once daily for 3 consecutive days following antibiotic administration, and twice weekly thereafter for 59 days. Male and female mice were placed together overnight at a 1:2–3 ratio following 6 weeks of fecal supernatant administration. Pregnancy was confirmed by the presence of vaginal spermatozoa. Mice that carried BPA and CON groups-derived microbiota (*n* = 10/group) were classified as GMT (BPA) and GMT (CON) groups, respectively. Each mouse was given water and an adequate standard diet [30].

For mice used to collect gut microbiota, gavage of microbial supernatants was conducted once daily at 0.2 mL/mouse. All pregnant mice from both groups were euthanized on day 18 of gestation. Placental and fetal weights were recorded in both mice groups. Later, blood, placental, ileal, and colonic samples were collected for respective parameter measurements [31, 32].

### Gut morphological analysis

Ileal samples were first fixed using 4% paraformaldehyde, then washed by gradient concentrations of ethanol and xylene for drying, embedded in paraffin, and processed into 5-μm sections. Thereafter, the 5-μm sections were deparaffined and rehydrated with gradient concentrations of ethanol and xylene. For investigating gut morphology, the hematoxylin–eosin stain was added to slides with 20 well-oriented crypts and villi from each section in every slide (Optimus software, version 6.5, Media Cybergenetics). Later, we calculated the villi/crypt ratio (VCR). Villus length and crypt depth were then quantified using at least 5 villi and crypts from every slide [33].

### Total antioxidant capacity (T-AOC), glutathione peroxidase (GSH-Px), superoxide dismutase (SOD), and malondialdehyde (MDA) activities

Activities of GSH-Px (cat. no. A005-1), SOD (cat. no. A001-3), T-AOC (cat. no. A005-1) and MDA (cat. no. A003-1) within plasma, ileum, and placenta were detected by associated assay kits (Nanjing Jiancheng Bioengineering Institute, Nanjing, China) in line with prior reports [7, 34]. Every assay was conducted six times.

### Caspase-3, caspase-8, and caspase-9 activities and mitochondrial cytochrome c level within placentome or intestine

The caspases-3 (cat. no. G015-1), caspase-8 (cat. no. G017-1), and caspase-9 (cat. no. G018-1) levels were measured with colorimetric assay kits (Nanjing Jiancheng Bioengineering Institute, Nanjing, China). For measuring mitochondrial cytochrome c level, approximately 0.10 g placentome or ileum was homogenized and lysed with pre-chilled lysis solution (50 μL). The cell lysates were centrifuged for 5-min at 10,000 × g and 4 ℃, and then 50 μL supernatant aliquots were examined according to specific protocols. The 10% ileum or placentome homogenate was centrifuged at 2000 × *g* and 4 ◦C for 10 min to collect the supernatants into the tube for further centrifugation at 10,000 × *g* and 4 ℃. Thereafter, the resulting pellet was processed by resuspension within 1.5 mL of the pre-chilled lysis solution and later lysis for determining cytochrome c level using the NanoDrop spectrophotometer (WFJ 2100; UNIC Instrument). The calibration curve was plotted using bovine cytochrome C [7].

### Mitochondrial isolation, ROS, mitochondria membrane potential (ΔΨm) as well as adenosine triphosphate (ATP) measurements

Placenta or ileum tissue samples were processed into pieces within 10 volumes of cold Buffer A (consisting of 225 mM mannitol, 75 mM sucrose, 10 mM HEPES, 1 mg/mL fatty acid-free BSA, along with 0.1 mM EGTA, pH 7.4) that contained protease/phosphatase inhibitors (Roche). The tissues were homogenized using the Teflon dounce homogeniser (1500 rpm, 4 strokes), and mitochondria were isolated by differential centrifugation (for 15 min at 700 × *g* and 10,000 × *g*, respectively under 4 ◦C). Mitochondria-containing pellets were then resuspended into Buffer B (395 mM sucrose, 10 mM HEPES, 0.1 mM EGTA, pH 7.4) [32].

The ROS levels generated by placental or ileal mitochondria were analyzed following the incubation of mitochondrial pellets into 2 μmol/L 2′,7′-dichlorohydrofluorescein diacetate under 24 ◦C for 24 min. Fluorescence intensity was detected with the fluorescence microplate reader in line with specific instructions [35].

The ΔΨm was assayed using a specific kit (cat. no. C2008S, Beyotime Institute of Biotechnology, Shanghai, China) while the alterations in placental or ileal chondriosome ΔΨm were assessed with the microplate reader (FLx800, Bio-Tek Instruments Inc.) according to fluorescence emission type. Under the high mitochondrial ΔΨm, JC-1 monomers aggregated within the mitochondrial matrix to display red fluorescence (OD590 nm), by contrast, they gave green fluorescence (OD529 nm) in the case of low mitochondrial ΔΨm [31].

The ATP levels in placental or ileal mitochondria were determined by the ATP assay kit (cat. no. S0026, Beyotime Institute of Biotechnology, Shanghai, China) following a previously described protocol [36]. Data were expressed as fold changes in comparison with the CON group.

### Mitochondrial complexes I–IV activities within placental or ileal mitochondria

The Ultrasonic Processor (Branson, MO, USA) was employed for on-ice ultrasonication, and mitochondrial lysis was carried out 30 times for 3 s at 200 W at intervals of 10 s. The resulting lysate was processed through 15-min centrifugation at 12,000 × *g* and 4 ℃. The Bicinchoninic Acid protein assay kit (cat. no. A045-3, Nanjing Jiancheng Bioengineering Institute, Nanjing, China) was utilized for measuring protein content. Colorimetric kits (Shanghai Enzyme-linked Biotechnology Co., Ltd., Shanghai, China) were employed for measuring activities of mitochondrial complexes I (cat. no. ml092752), II (cat. no. ml092753), III (cat. no. ml092754), and IV (cat. no. ml092755) (NADH ubiquinone reductase, succinate ubiquinone reductase, ubiquinol cytochrome c reductase, together with cytochrome c oxidase) [37]. Data were normalized against total protein contents within every sample.

### Colonic lipopolysaccharide (LPS) and volatile fatty acids (VFAs) concentration

The LPS level within colonic digesta of mice (cat. no. CB10838-Mu) and ewes (cat. no. CB10063-Sp) was determined with the ELISA Kit (COIBO BIO, Shanghai, China) [38]. The VFAs (isobutyrate, acetate, isovalerate, propionate, butyrate, as well as valerate) contents within colonic digesta were determined by gas chromatography (14B, Shimadzu, Kyoto, Japan) using the film-thick capillary column of 30 m × 0.32 mm × 0.25 mm at the injection, column and detector temperatures of 180 ℃, 110 ℃, and 180 ℃, respectively, with crotonic acid as internal standard [39]. More details about VFAs assay have been described by Mass et al. [40].

### Gut microbiota examination

Total genomic DNA was extracted from colonic contents using primers with corresponding barcodes (16S V3 + V4) before amplification. Paired-end sequencing was completed using the Illumina MiSeq platform, while the phylogenic tree and OUT table were obtained using the Mothur Bayesian classifier. Thereafter, this work generated and analyzed sequencing libraries according to the prior description [41, 42]. The principal coordinates were obtained by conducting the principal coordinate analysis (PCoA). We performed PCoA based on unweighted UniFrac metrics to assess the differences in colonic microbiota between the two groups. Later, Simpson, Shannon, ACE, and Chao1 indexes of those detected species were determined to evaluate species diversity [43]. We used OTUs to predict the genome of microbial communities based on PICRUSt (Phylogenetic Investigation of Communities by Reconstruction of Unobserved States) [44].

### RT‑qPCR

The total RNA in placental or ileal tissues was extracted by TRIzol (Takara Bio, Otsu, Japan). Then, RNA (1 μg) was collected to prepare cDNA using PrimeScript® RT Reagent Kit with cDNA Eraser (Takara Bio, Otsu, Japan) through reverse transcription. The ABI 7300 real-time PCR system (Applied Biosystems, Foster, CA, USA) was adopted for RT-qPCR using the SYBR Green master mix with gene-specific primers (Table S2). The cycle threshold (Ct) approach was applied to determine relative fold changes in gene levels, with β-actin being the endogenous control [45].

### Western blotting

Western blotting of the total proteins extracted from placental or ileal tissues was conducted in line with a previous protocol [46]. In brief, placental or ileal tissues were homogenized with the tissue protein extract (Thermo Fisher Scientific, USA) and then centrifuged at 12,000 × *g* and 4 °C for 10 min to obtain the supernatants. The protein contents in these supernatants were measured using the BCA Protein Assay Kit (Thermo Fisher Scientific, USA). Targeted proteins were separated through 12% SDS-PAGE based on molecular size and then transferred onto the 0.45-μm PVDF membranes. Afterward, membranes were blocked using 5% defatted milk under ambient temperature for 3 h. Additionally, corresponding primary antibodies for antioxidant, apoptosis, ERS, autophagy, and voltage-dependent anion channel (VDAC) or β-actin were introduced for overnight incubation under 4 °C. Following 2-h incubation using goat anti-rabbit IgG secondary antibodies under ambient temperature, the ECL plus western blotting detection system was employed for protein detection. Protein expression was standardized to VDAC or β-actin expression and normalized to the mean ± SEM of the CON group. Table S3 presents more details about antibodies.

### Statistical analysis

Homogeneity of variance was tested for the measures with Levene’s test. The data were tested between the 2 groups for significance with an independent sample *t* test method, with SPSS 17.0 (SPSS, Chicago, IL, USA). Data were represented by mean ± SEM. *P* < 0.05 stood for statistical significance. Bivariate correlation analysis in SPSS 17.0 was used to calculate the Spearman correlation coefficients between the key gut bacteria at the genus level and other measures. *P* < 0.05 was used to identify a significant correlation.

## Results

### Effect of BPA exposure on placental weight, fetal weight, placental apoptosis, OS and mitochondrial dysfunction of pregnant ewes

Maternal BPA exposure induced apoptosis, autophagy, ERS, OS, and mitochondrial dysfunction in the ovine placenta, and decreased the total weights of type A placentomes, fetal weight, and placental efficiency [7].

### Effects of GMT from donor pregnant Hu ewes in CON and BPA groups on placental weight, fetal weight, placental antioxidant and apoptosis-related enzyme activity and mitochondrial function of antibiotics-treated mice

**Fig. 1:**
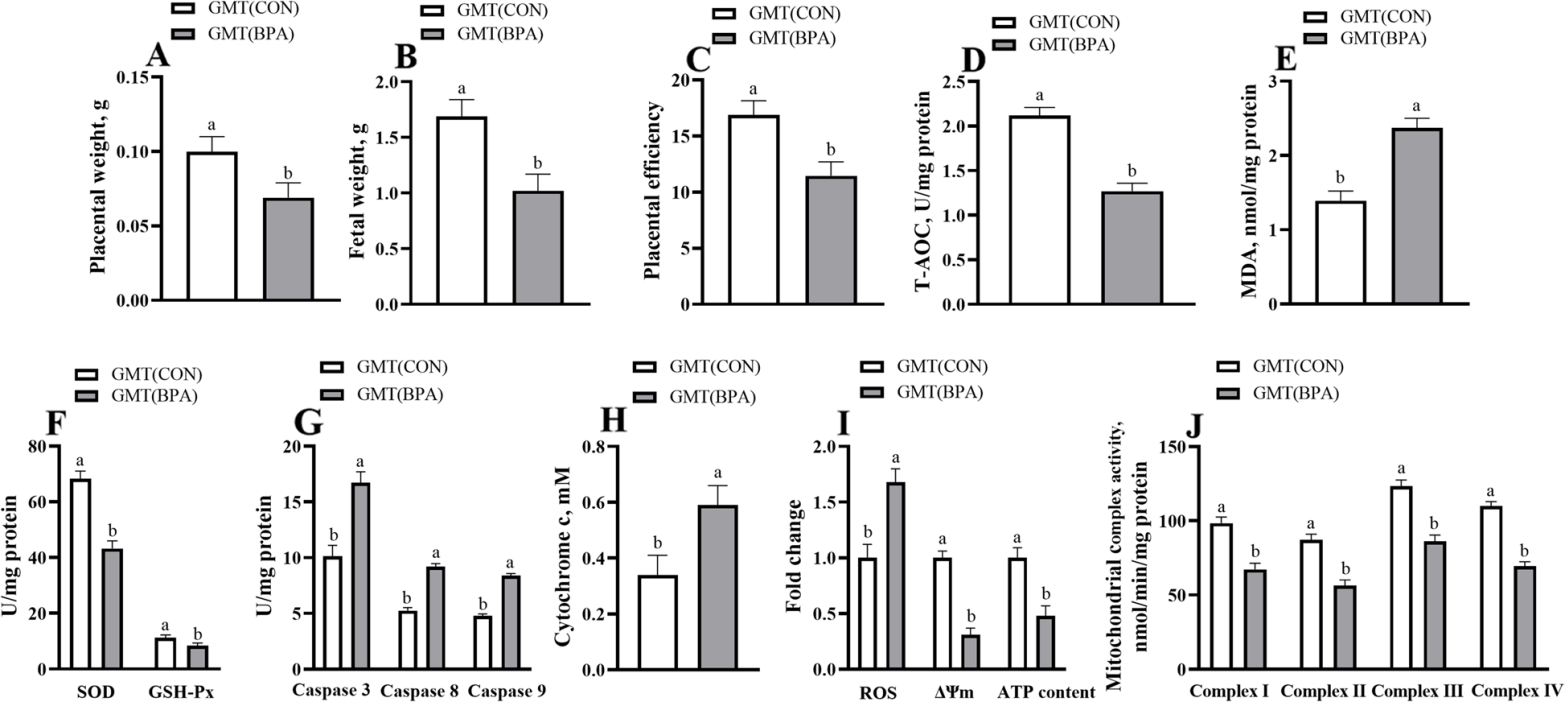
Effects of GMT from donor pregnant Hu sheep in CON and BPA groups on placental weight, fetal weight, placental efficiency, placental antioxidant and apoptosis-related enzyme activity and mitochondrial function in antibiotics-treatment mice on day18 of gestation. **A** Placental weight. **B** Fetal weight. **C** Placental efficiency. Placental efficiency is calculated as the ratio of fetal weight/total placental weight. **D** The T-AOC activity. **E** The MDA activity. **F** GSH-Px and SOD activities. **G** Caspase 3, caspase 8, and caspase 9 activities. **H** The cytochrome c concentration. **I** ROS, ΔΨm, and ATP levels. **J** Mitochondrial complex activity. The data are shown as mean ± SEM, *n* = 8. Different lowercase letters represent significant differences at *P* < 0.05

The GMT (BPA) mice experienced a reduction (*P* < 0.05) in placental weight, fetal weight, placental efficiency, T-AOC, GSH-Px, and SOD activities in the placenta, placental ΔΨm, ATP level, and activities of mitochondrial complexes I-IV in comparison with GMT (CON) mice (Fig. 1). Nonetheless, Caspase 3, Caspase 8, Caspase 9 activities, and MDA, Cytochrome c, and ROS levels within the placenta of GMT (BPA) mice were elevated (*P* < 0.05) compared to those of the GMT (CON) mice.

### Impact of GMT from donor pregnant Hu ewes in CON and BPA groups on antioxidation, apoptosis, autophagy and ERS-associated gene, and protein levels in placental tissue of antibiotics-treated mice from day 0 to day 18 of gestation

**Fig. 2:**
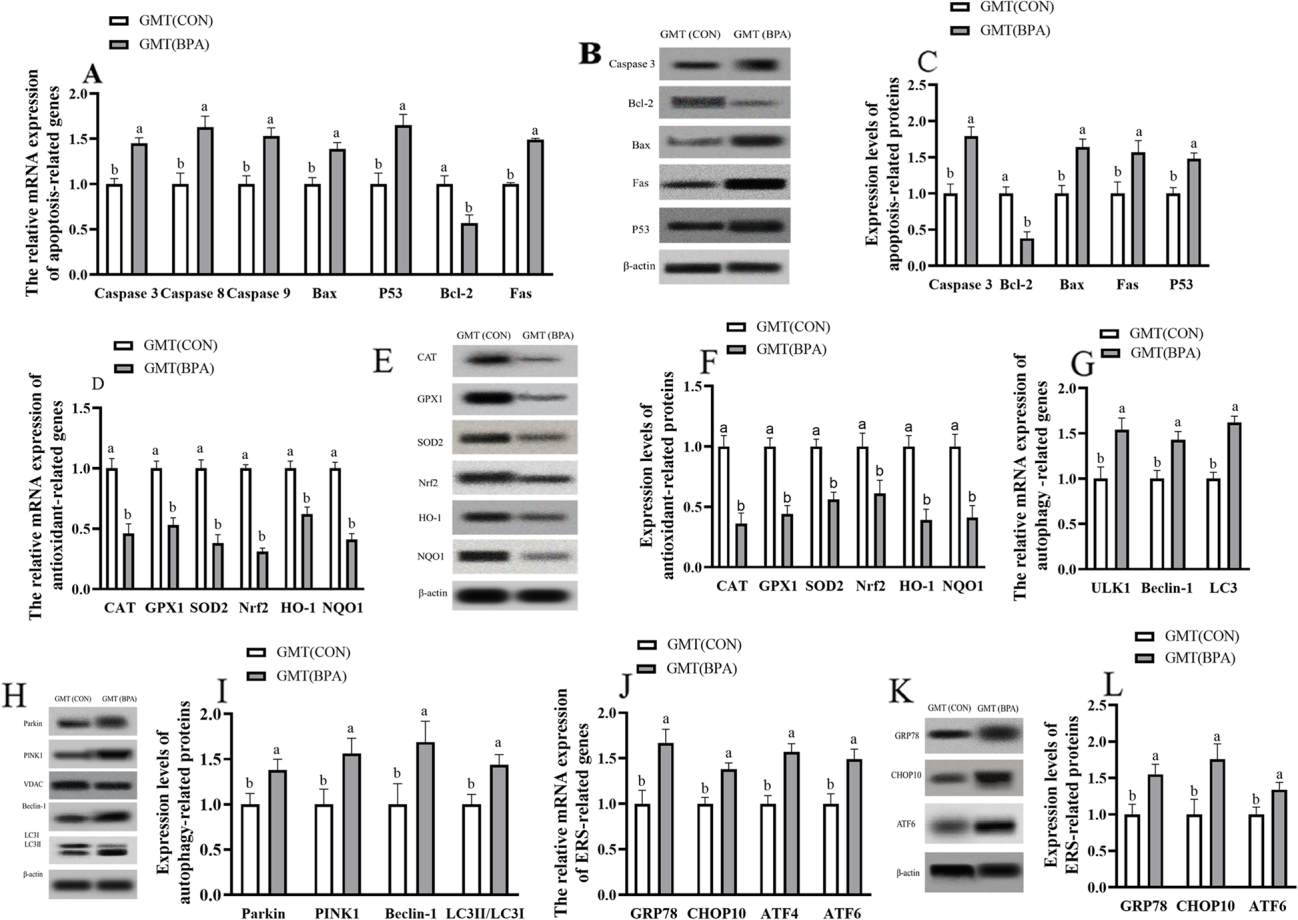
Effects of GMT from donor pregnant Hu sheep in CON and BPA groups on the mRNA and protein relative expressions of antioxidation, apoptosis, autophagy, and ERS in placental tissue in antibiotics-treatment mice on day 18 of gestation. **A** The mRNA abundance of apoptosis-related genes. **B** Representative immunoblots of apoptosis-related proteins. **C** Expression levels of apoptosis-related proteins. **D** The mRNA abundance of antioxidant-related genes. **E** Representative immunoblots of antioxidant-related proteins. **F** Expression levels of antioxidant-related proteins. **G** The mRNA abundance of autophagy-related genes. **H** Representative immunoblots of autophagy-related proteins. **I** Expression levels of autophagy-related proteins. **J** The mRNA abundance of ERS-related genes. **K** Representative immunoblots of ERS-related proteins. **L** Expression levels of ERS-related proteins. All the relative expression levels of mRNA and protein were normalized to the β-actin and were expressed relative to those of the GMT (CON) group (fold of GMT (CON)). The data are shown as mean ± SEM, *n* = 10. Different lowercase letters represent significant differences at *P* < 0.05

The antioxidation and apoptosis-associated gene and protein expression (GPx1, CAT, SOD2, HO-1, Nrf2, NQO1, and Bcl 2) were decreased (*P* < 0.05), but apoptosis-related genes and proteins (P53, Fas, Bax, and caspase 3), autophagy-related genes (Beclin1, ULK1, and LC3) and proteins (Parkin, PINK1, Beclin1, and LC3II/3I), ERS-related genes and proteins (GRP78, CHOP10, and ATF6) were increased (*P* < 0.05) in placenta of GMT (BPA) mice compared to those in GMT (CON) mice (Fig. 2).

### Maternal ileal morphology, antioxidant and apoptosis-related enzyme activity, and mitochondrial function in ewes and mice during pregnancy

**Fig. 3:**
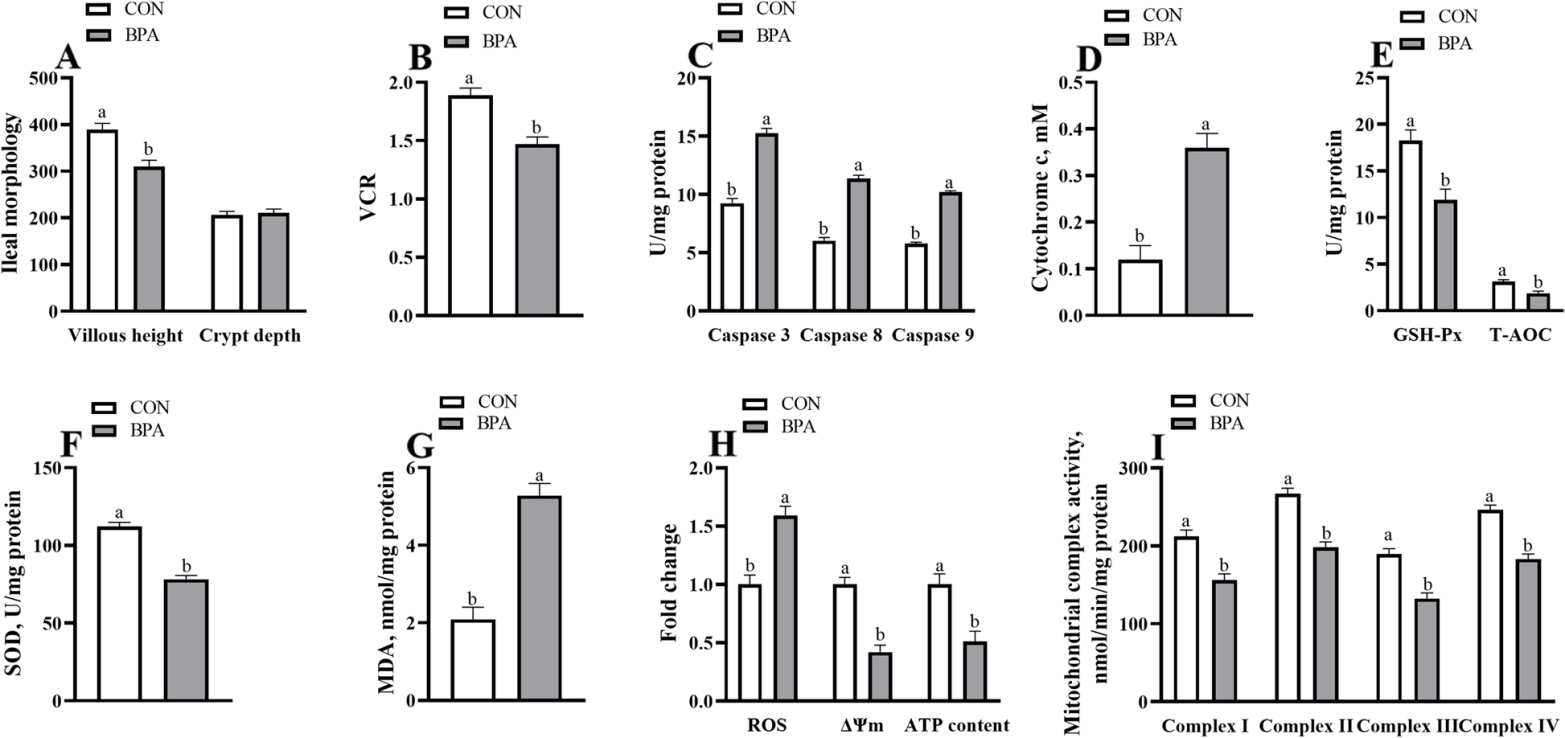
Effect of BPA exposure on maternal ileal morphology, antioxidant, and apoptosis-related enzyme activity, and mitochondrial function in pregnant ewes on day 110 of gestation. **A** Villus morphology. **B** VCR. **C** Caspase 3, caspase 8, and caspase 9 activities. **D** The cytochrome c concentration. **E** GSH-Px and T-AOC activities. **F** The SOD activity. **G** The MDA activity. **H** ROS, ΔΨm, and ATP levels. **I** Mitochondrial complex activity. The data are shown as mean ± SEM, *n* = 8. Different lowercase letters represent significant differences at *P* < 0.05

**Fig. 4:**
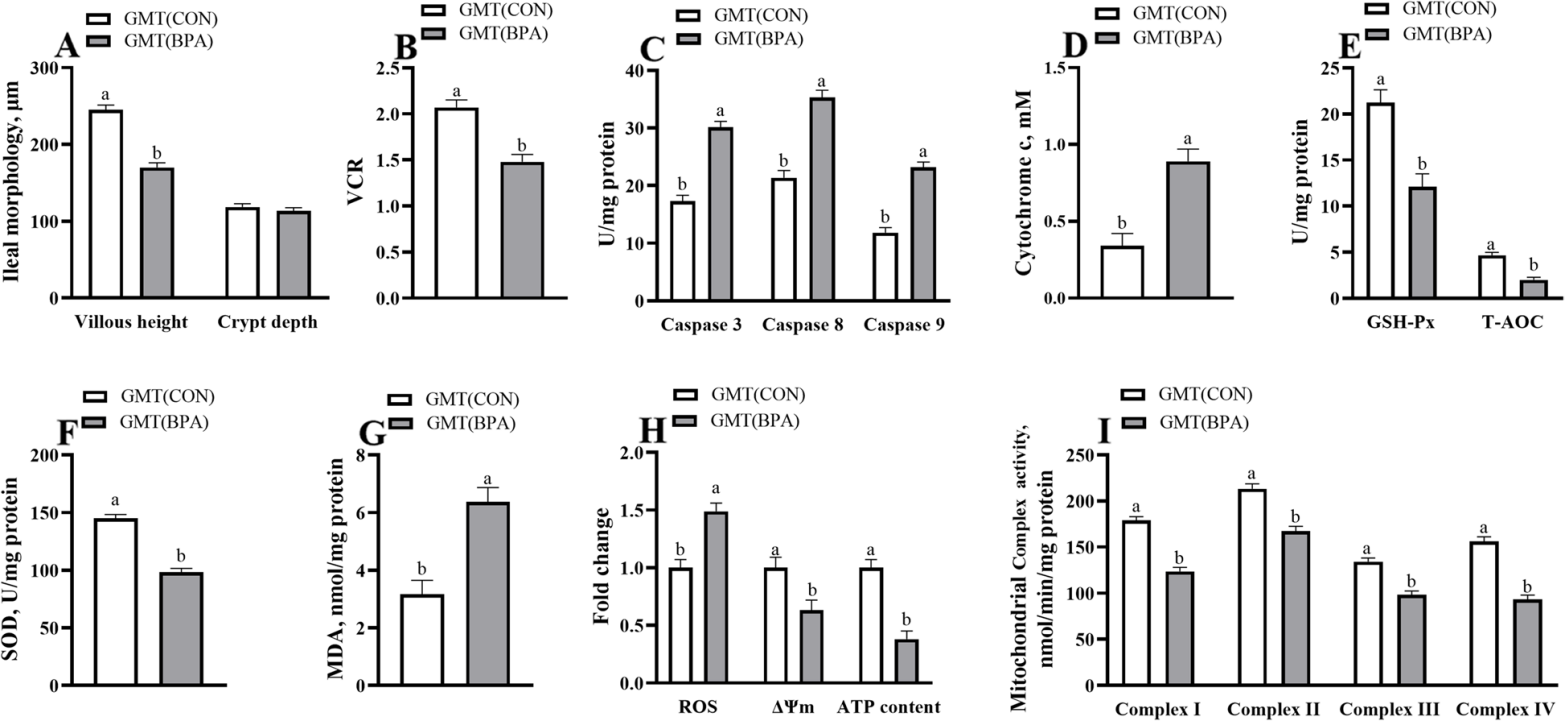
Effects of GMT from donor pregnant Hu sheep in CON and BPA groups on maternal ileal morphology, antioxidant, and apoptosis-related enzyme activity, and mitochondrial function in antibiotics-treated mice on day 18 of gestation. **A** Villus morphology. **B** VCR. **C** Caspase 3, caspase 8, and caspase 9 activities. **D** The cytochrome c concentration. **E** GSH-Px and T-AOC activities. **F** The SOD activity. **G** The MDA activity. **H** ROS, ΔΨm, and ATP levels. **I** Mitochondrial complex activity. The data are shown as mean ± SEM, *n* = 10. Different lowercase letters represent significant differences at *P* < 0.05

The BPA ewes or GMT (BPA) mice showed a decrease in maternal ileal epithelium integrity, villous height, VCR, T-AOC, SOD, and GSH-Px activities, and ileal ΔΨm, ATP levels, and mitochondrial complexes I–IV activities (*P* < 0.05) compared to those in CON ewes or GMT (CON), respectively (Fig. S1; Figs. 3 and 4). Nonetheless, BPA ewes or GMT (BPA) mice have elevated Caspase 3, Caspase 8, and Caspase 9 activities, and MDA, Cytochrome c level, and ROS production in the ileum (*P* < 0.05) relative to CON ewes or GMT (CON) mice, respectively.

### Antioxidation, apoptosis, autophagy and ERS-related gene, and protein levels of maternal ileum in ewes and mice during pregnancy

**Fig. 5:**
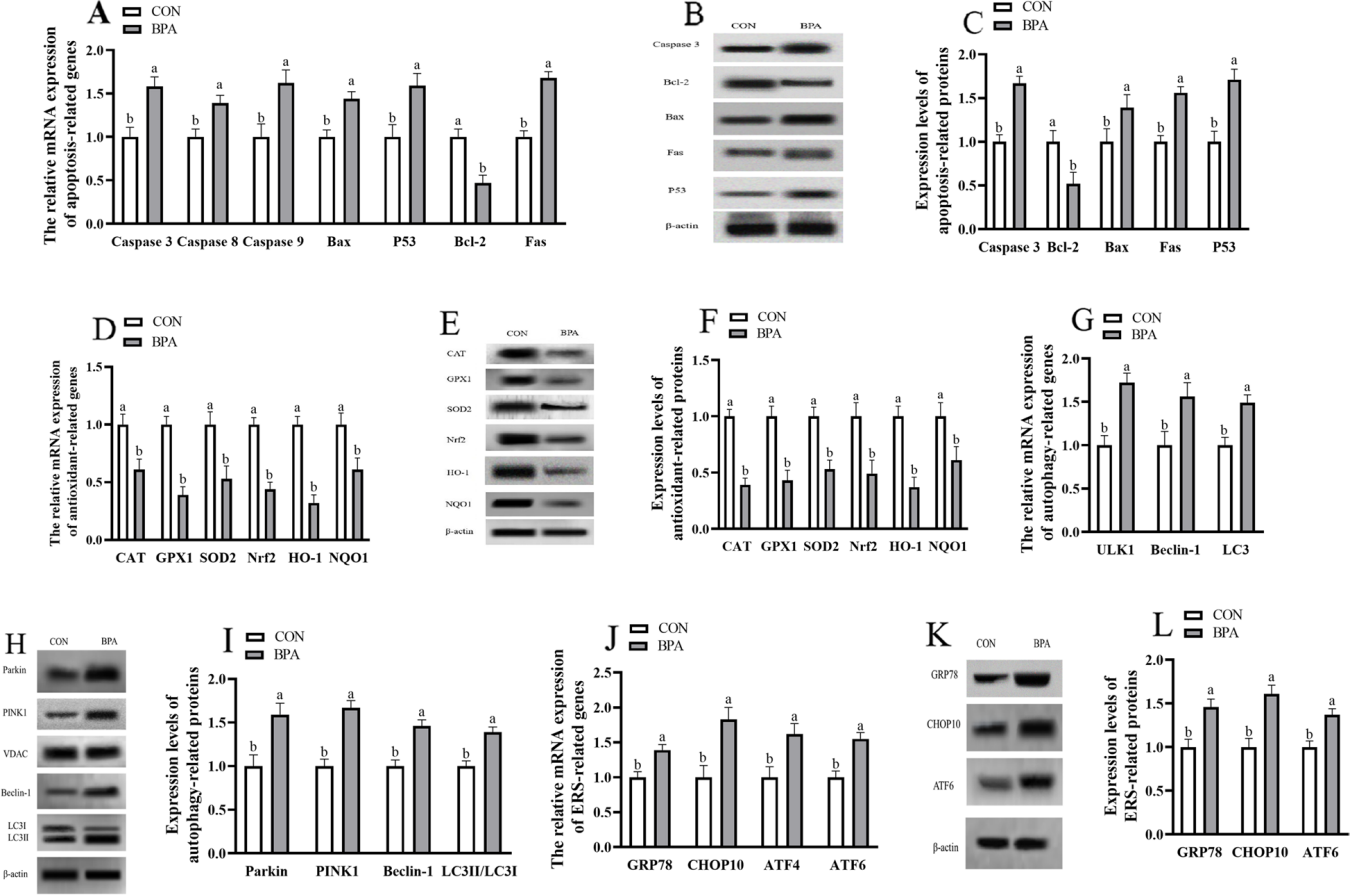
Effect of BPA exposure on the mRNA and protein relative expressions of apoptosis, antioxidation, autophagy and ERS in maternal ileum in pregnant ewes on day 110 of gestation. **A** The mRNA abundance of apoptosis-related genes. **B** Representative immunoblots of apoptosis-related proteins. **C** Expression levels of apoptosis-related proteins. **D** The mRNA abundance of antioxidant-related genes. **E** Representative immunoblots of antioxidant-related proteins. **F** Expression levels of antioxidant-related proteins. **G** The mRNA abundance of autophagy-related genes. **H** Representative immunoblots of autophagy-related proteins. **I** Expression levels of autophagy-related proteins. **J** The mRNA abundance of ERS-related genes. **K** Representative immunoblots of ERS-related proteins. **L** Expression levels of ERS-related proteins. The data are shown as mean ± SEM, *n* = 8. Different lowercase letters represent significant differences at *P* < 0.05. All the relative expression levels of mRNA and protein were normalized to the β-actin and were expressed relative to the CON group (fold of CON)

**Fig. 6:**
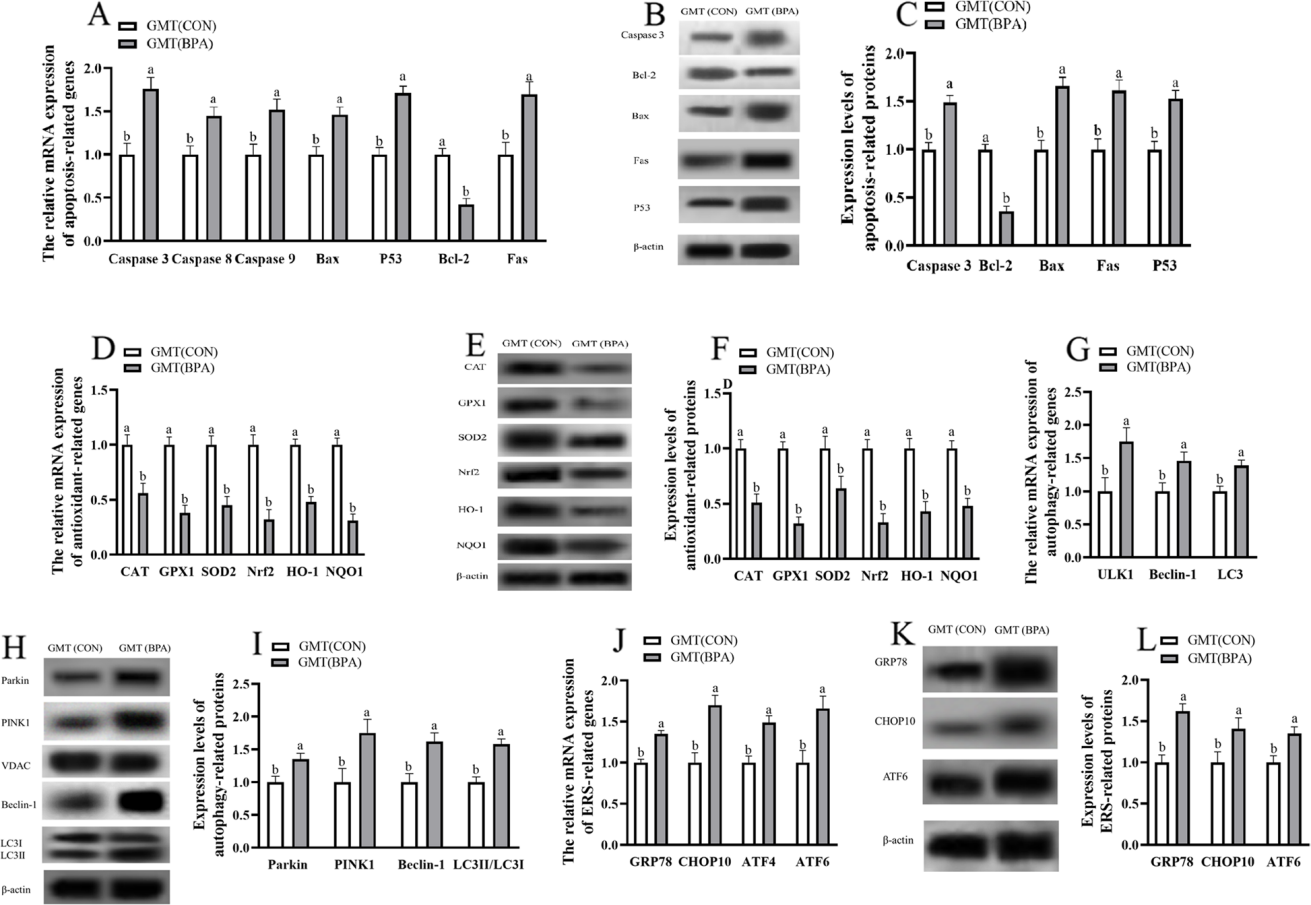
Effects of GMT from donor pregnant Hu sheep in CON and BPA groups on the mRNA and protein relative expressions of antioxidation, apoptosis, autophagy, and ERS in maternal ileum in antibiotics-treated mice on day 18 of gestation. **A** The mRNA abundance of apoptosis-related genes. **B** Representative immunoblots of apoptosis-related proteins. **C** Expression levels of apoptosis-related proteins. **D** The mRNA abundance of antioxidant-related genes. **E** Representative immunoblots of antioxidant-related proteins. **F** Expression levels of antioxidant-related proteins. **G** The mRNA abundance of autophagy-related genes. **H** Representative immunoblots of autophagy-related proteins. **I** Expression levels of autophagy-related proteins. **J** The mRNA abundance of ERS-related genes. **K** Representative immunoblots of ERS-related proteins. **L** Expression levels of ERS-related proteins. The data are shown as mean ± SEM, *n* = 10. Different lowercase letters represent significant differences at *P* < 0.05. All the relative expression levels of mRNA and protein were normalized to the β-actin and were expressed relative to the GMT (CON) mice (fold of GMT (CON))

The antioxidation- and apoptosis-related genes and proteins (GPx1, CAT, SOD2, HO-1, NQO1, Nrf2, and Bcl 2) levels were lower (*P* < 0.05), but apoptosis-related genes and proteins (P53, Fas, Bax, and caspase 3), autophagy-related genes (ULK1, Beclin1, and LC3) and proteins (PINK1, Parkin, Beclin1, and LC3II/3I), ERS-related genes and proteins (GRP78, CHOP10, and ATF6) levels were higher (*P* < 0.05) in maternal ileum of BPA ewes or GMT (BPA) mice relative to those in CON ewes or GMT (CON) mice, respectively (Figs. 5 and 6).

### Maternal colonic LPS and VFAs concentrations in ewes and mice during pregnancy

**Fig. 7:**
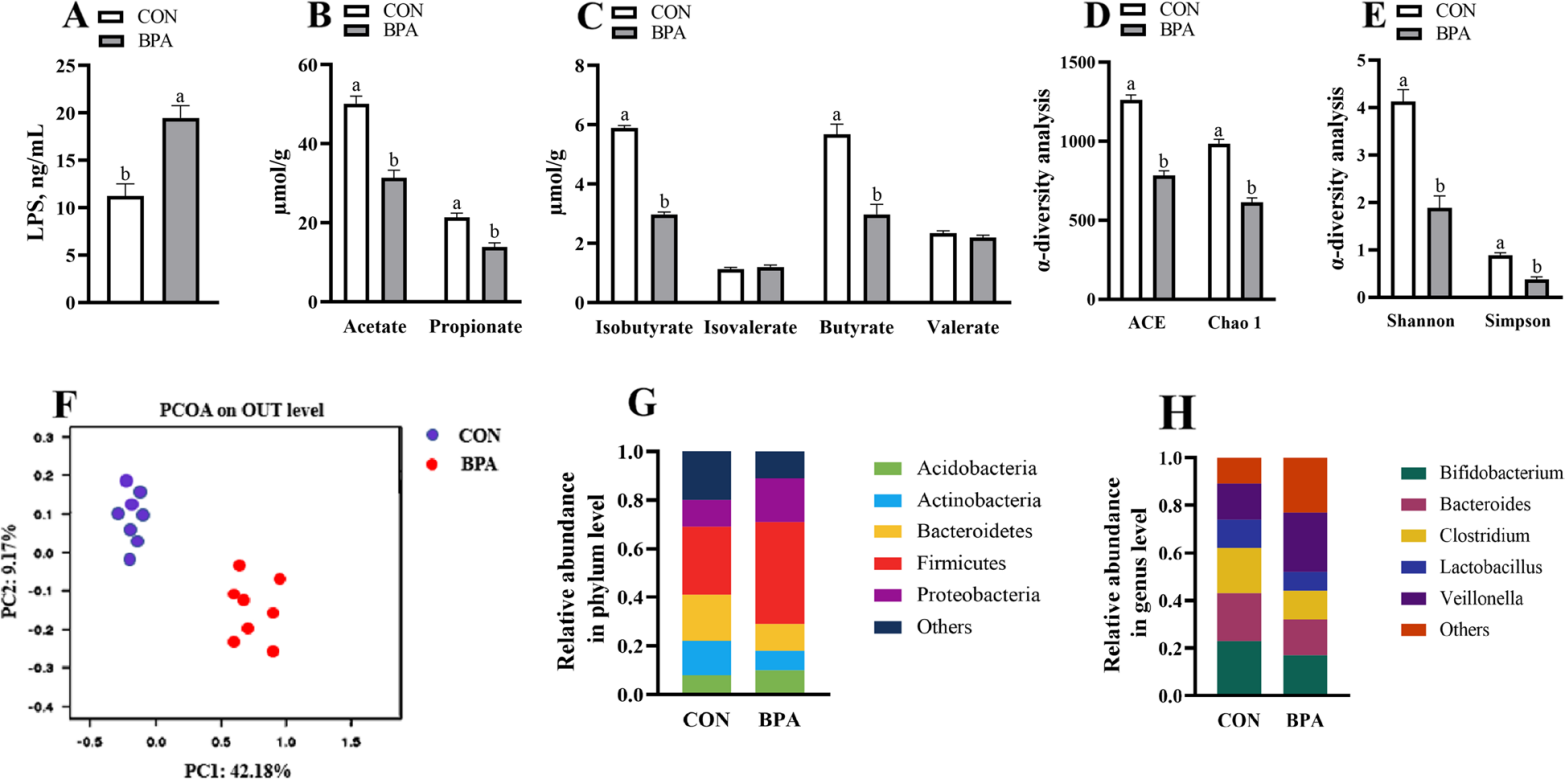
Effect of BPA on maternal colonic LPS concentrations, VFAs, and microbiota in pregnant Hu sheep on day 110 of gestation. **A** LPS concentrations. **B** Concentrations of propionate and acetate. **C** Concentrations of butyrate, valerate, isobutyrate, and isovalerate. **D** ACE and Chao1. **E** Shannon and Simpson. **F** PCoA plots (unweighted UniFrac) of bacterial communities, based on OTUs. **G** Microbiota compositions at the phylum level. **H** Microbiota compositions at the genus level. The data are shown as mean ± SEM, *n* = 8. Different lowercase letters represent significant differences at *P* < 0.05

**Fig. 8:**
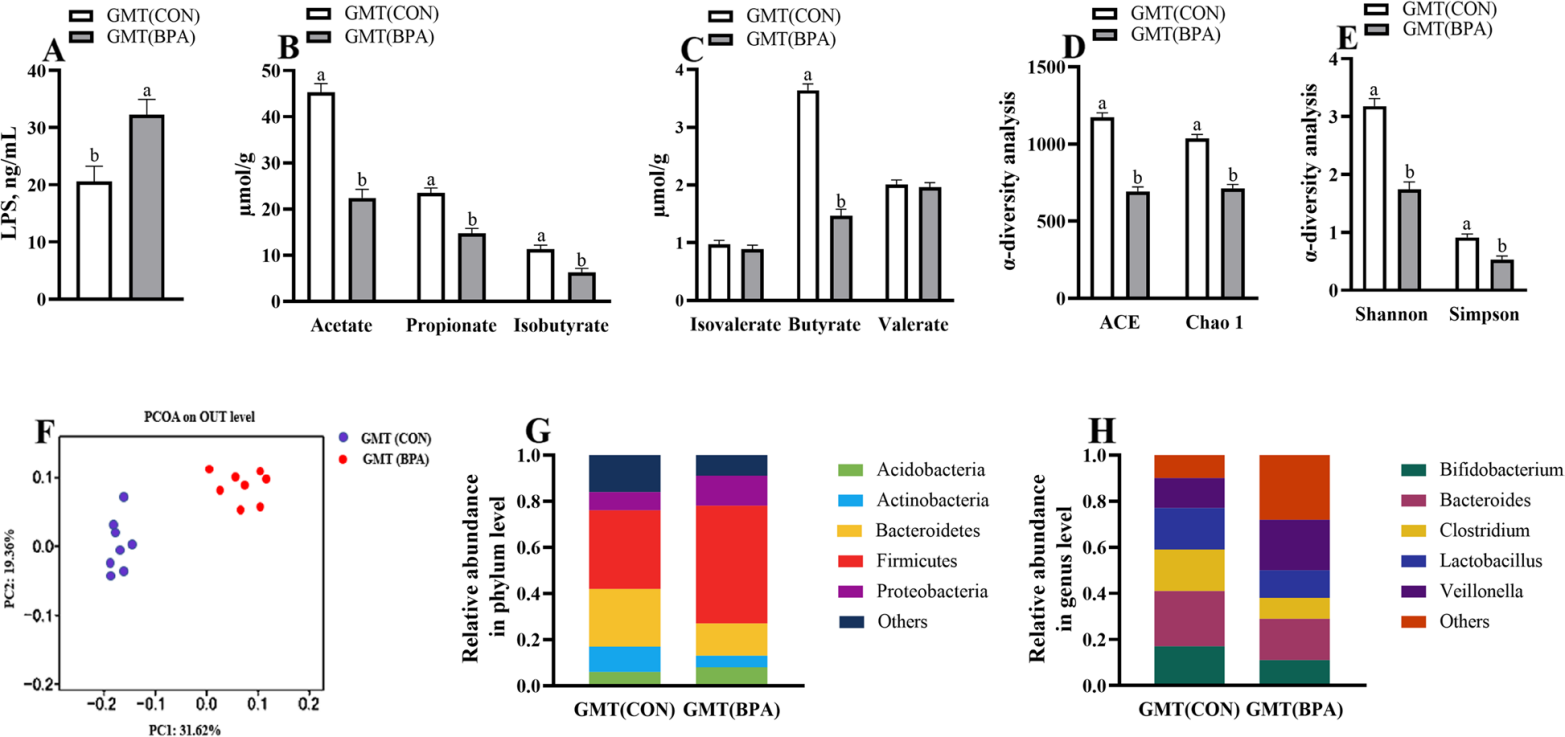
Maternal colonic LPS concentrations, VFAs, and microbiota of pregnant mice responded to gut microbiota transplant from CON and BPA pregnant ewes. **A** LPS concentrations. **B** Concentrations of propionate, isobutyrate, and acetate. **C** Concentrations of butyrate, valerate, and isovalerate. **D** ACE and Chao1. **E** Shannon and Simpson. **F** PCoA plots (unweighted UniFrac) of bacterial communities, based on OTUs. **G** Microbiota compositions at the phylum level. **H** Microbiota compositions at the genus level. The data are shown as mean ± SEM, *n* = 8. Different lowercase letters represent significant differences at *P* < 0.05

The acetate, butyrate, propionate, and isobutyrate levels were decreased (*P* < 0.05), while LPS level was increased (*P* < 0.05) within the colonic contents in BPA ewes or GMT (BPA) mice relative to those in the CON ewes or GMT (CON) mice, respectively (Figs. 7A–C and 8A–C).

### Maternal colonic microbiota in ewes and mice during pregnancy

The BPA ewes or GMT (BPA) mice exhibited a decline (*P* < 0.05) in ACE, Chao1, Simpson, and Shannon indexes compared to those of the CON ewes or GMT (CON) mice, respectively (Figs. 7D, E and 8D, E). Obvious clustering of microbial compositions was observed in pregnant CON and BPA ewes (Fig. 7F) or pregnant GMT (CON) and GMT (BPA) mice (Fig. 8F). Actinobacteria and Bacteroidetes abundances decreased whereas Proteobacteria and Firmicutes abundances, and Firmicutes/Bacteroidetes ratio increased (*P* < 0.05) at phylum level in BPA ewes or GMT-(BPA) mice relative to those in CON ewes or GMT (CON) mice, respectively (Figs. 7G and 8G). Bifidobacterium, Bacteroides, Clostridium, and Lactobacillus showed decreased abundances at genus level (*P* < 0.05), whereas Veillonella increased abundance (*P* < 0.05) in the BPA ewes or GMT (BPA) mice relative to those in the CON ewes or GMT (CON) mice, respectively (Figs. 7H and 8H).

### Correlation analysis between the key gut bacteria at the genus level and other measures

**Fig. 9:**
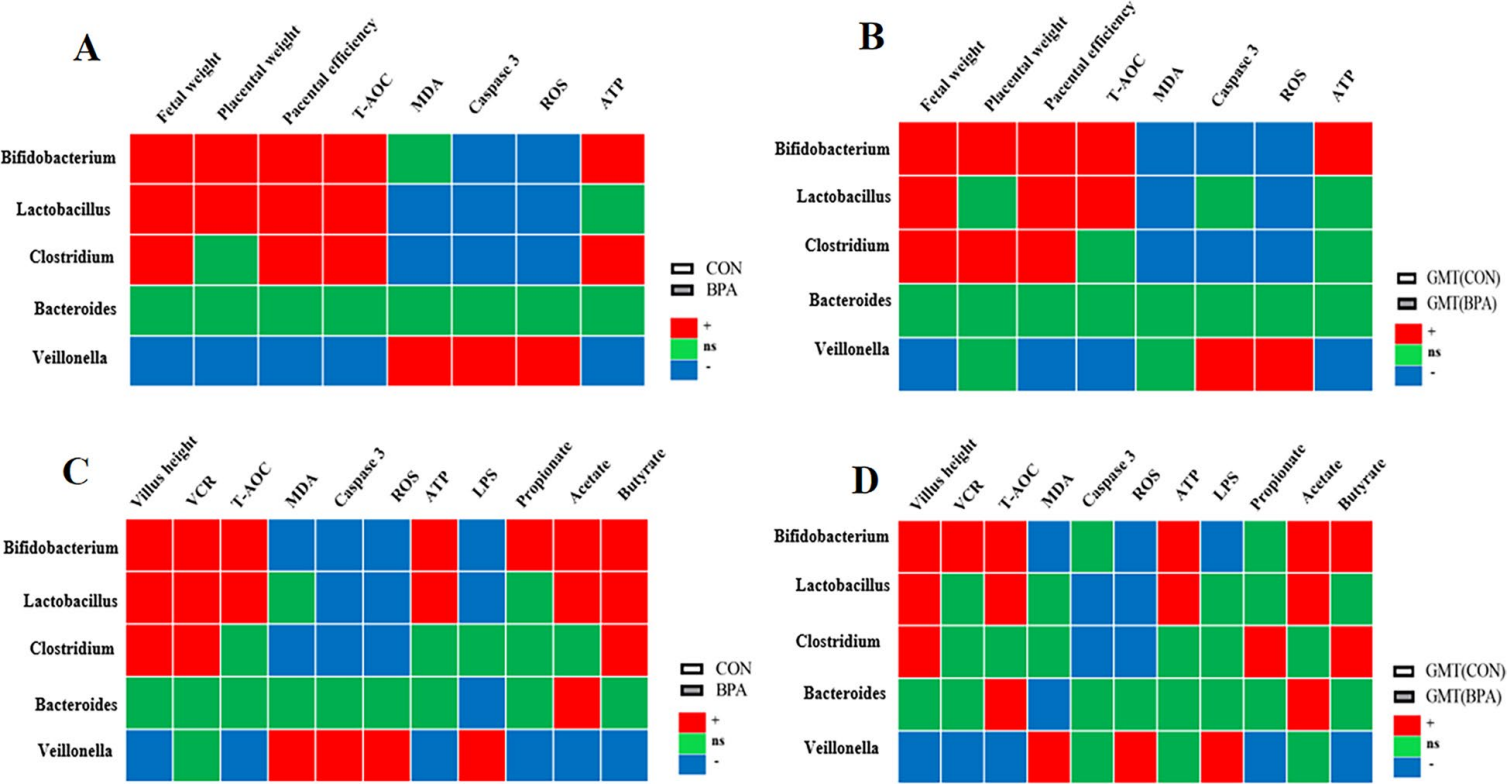
Correlation analysis between the key gut bacteria at the genus level and other measures. **A** Spearman correlation between the key gut bacteria at the genus level and fetal weight, placental weight, placental efficiency, the activities of T-AOC, MDA, and caspase 3, and the levels of ROS and ATP in cotyledon tissues in CON and BPA pregnant ewes. **B** Spearman correlation between the key gut bacteria at the genus level and fetal weight, placental weight, placental efficiency, the activities of T-AOC, MDA, and caspase 3, and the levels of ROS and ATP in the placenta in GMT (CON) and GMT (BPA) mice. **C** Spearman correlation between the key gut bacteria at the genus level and villus weight, VCR, the activities of T-AOC, MDA, and caspase 3, the levels of ROS, ATP and LPS, and VFAs concentration in intestinal tissues in CON and BPA pregnant ewes. **D** Spearman correlation between the key gut bacteria at the genus level and villus weight, VCR, the activities of T-AOC, MDA, and caspase 3, the levels of ROS, ATP and LPS, and VFAs concentration in intestinal tissues in GMT (CON) and GMT (BPA) mice. “ + ” represents a significantly positive correlation (*P* < 0.05), “ − ” represents a significantly negative correlation (*P* < 0.05), and “ns” represents non-significant correlation (*P* > 0.05). The fetal weight, placental weight, placental efficiency, the activities of T-AOC, MDA, and caspase 3, and the levels of ROS and ATP in cotyledon tissues in CON and BPA pregnant ewes have been published [7]

The relative abundances of Bifidobacterium, Lactobacillus, and Clostridium showed a significantly positive correlation with fetal weight, placental efficiency, and the T-AOC activity in cotyledon tissues (*P* < 0.05), while the relative abundances of Veillonella showed negative correlation with fetal weight, placental weight, placental efficiency, the T-AOC activity and ATP level in cotyledon tissues in CON and BPA pregnant ewes (*P* < 0.05) (Fig. 9A). The relative abundances of Bifidobacterium, Lactobacillus, and Clostridium showed a significantly positive correlation with placental efficiency and fetal weight (*P* < 0.05), while the relative abundances of Veillonella showed a negative correlation with fetal weight, placental efficiency, the T-AOC activity and ATP level in the placenta in GMT (CON) and GMT (BPA) mice (*P* < 0.05) (Fig. 9B).

The relative abundances of Bifidobacterium, Clostridium, and Lactobacillus showed a significantly positive correlation with villus height, VCR, and butyrate concentration (*P* < 0.05), while the relative abundances of Veillonella showed a negative correlation with villus height, the T-AOC activity, ATP level, and the concentrations of propionate, acetate and butyrate in intestinal tissues in CON and BPA pregnant ewes (*P* < 0.05) (Fig. 9C). The relative abundances of Bifidobacterium and Lactobacillus showed a significantly positive correlation with villus height, T-AOC activity, ATP level, and acetate concentration (*P* < 0.05), while the relative abundances of Veillonella showed a negative correlation with villus weight, VCR, T-AOC activity and the concentrations of propionate and butyrate in intestinal tissues in GMT (CON) and GMT (BPA) mice (*P* < 0.05) (Fig. 9D).

## Discussion

**Fig. 10:**
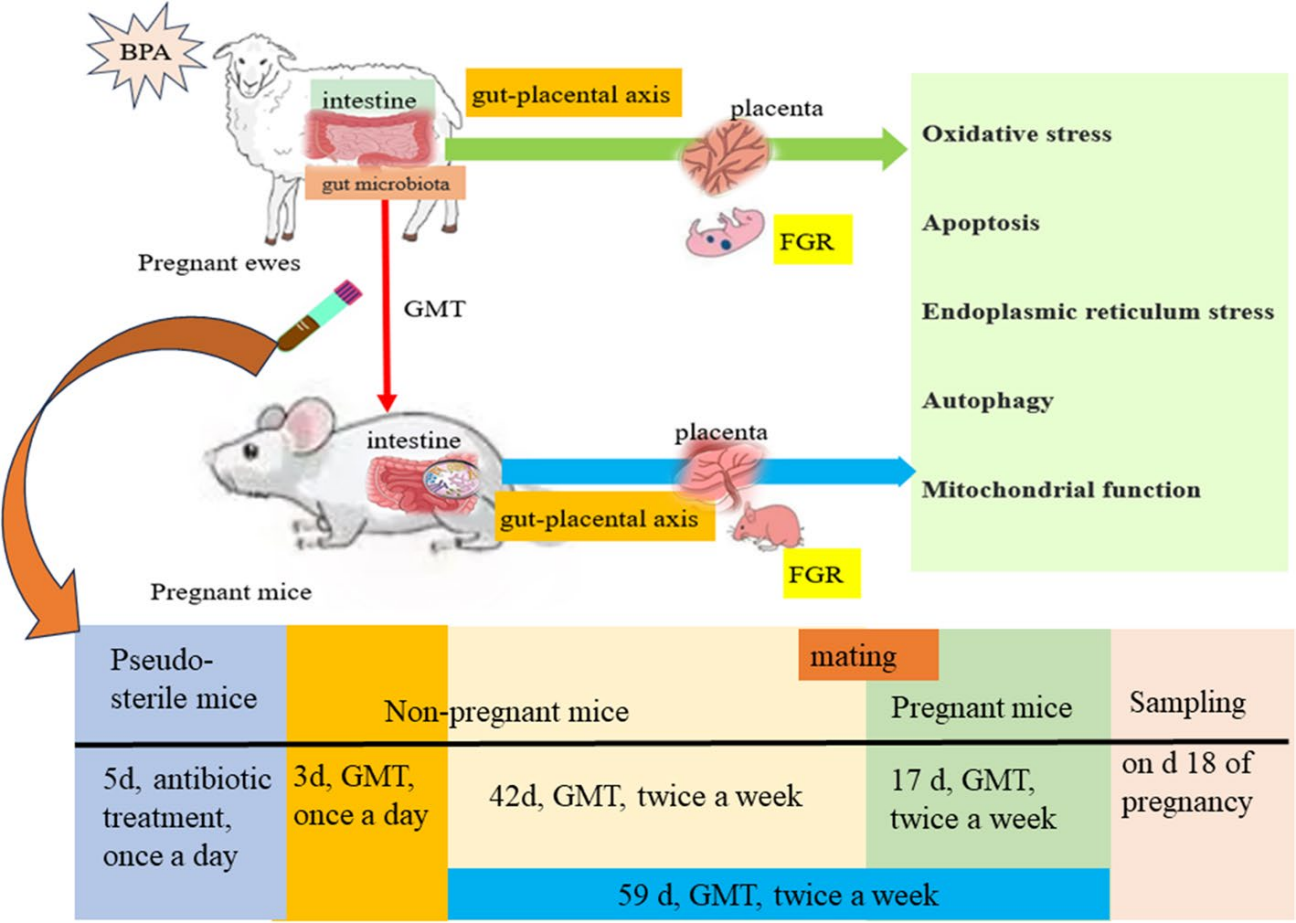
Graphical abstract. Gut microbiota contributes to the BPA-induced maternal intestinal and placental apoptosis, OS, and FGR in pregnant ewe by GMT trial of trans-species from sheep to mice

The FGR is associated with neonatal mortality as well as chronic diseases in adulthood [47]. BPA is an endocrine-disrupting chemical (EDC) with a high environmental distribution level, which can be detected within placental tissues. Its accumulation in the placenta induces placental dysfunctions and leads to FGR [1, 7]. Placental efficiency accounts for a critical factor reflecting the uterine capacity of an animal [48]. Placental efficiency indirectly measures the placental functioning for nutrient delivery to the fetus and serves as an important factor for determining the total fetal growth [49]. In our previous study, prenatal BPA exposure markedly decreased the total fetal weight on day 110 of gestation [7]. In our current study, the fetal weights of GMT (BPA) mice were reduced compared to those of GMT (CON) mice. Therefore, the BPA ewes or GMT (BPA) mice might have a reduced placental efficiency compared to the CON ewes or GMT (CON) mice, respectively. Our previous research also showed that maternal BPA exposure induced apoptosis, autophagy, ERS, OS, mitochondrial dysfunction, decreased placental efficiency in ovine placenta, and further resulted in FGR in pregnant ewes [7]. To further explore whether the BPA-evoked FGR in pregnant ewes was through modulating gut microbiota, fecal supernatants collected from CON and BPA pregnant ewes were transferred into microflora-free mice (Fig. 10). Transferring donor phenotypes to recipients is necessary to indicate the link between microbiota modulation and disease outcomes by using fecal microbiota transplantation (FMT) [50, 51]. Furthermore, cross-species FMT from sheep to mice is feasible and has been verified by various experiments [19, 52, 53]. In our study, the results showed that BPA mediated FGR through modulating gut microbiota.

Previous studies have shown that increased placental OS is associated with the occurrence of adverse pregnancy outcomes, including stillbirth and FGR [54]. Additionally, the mammal placenta has independent antioxidant systems like SOD, CAT and GSH-Px [31]. The OS triggers apoptosis while affecting cell homeostasis, and this may be related to the imbalance between anti-oxidation and pro-oxidation [55]. MDA, the metabolite generated via lipid peroxidation, is the predicting factor for OS and ROS [56]. In our study, the apparent decrease of antioxidation-related enzyme activity (GSH-Px, T-AOC, and SOD) and antioxidation-related gene and protein levels (CAT, GPX1, SOD2, Nrf2, HO-1, NQO1), and the elevated MDA levels within maternal placental and ileal tissues of BPA pregnant ewes verified the occurrence of OS. Moreover, antioxidant function in the placental and ileal tissues of GMT (BPA) mice was decreased relative to GMT (CON) mice. Consequently, OS of the placenta and ileum in BPA pregnant ewes may be aggravated through modulation of gut microflora.

Mitochondria are the cellular metabolic process centers, which execute their basic function to ensure the persistent physiological functions of trophoblast cells [57]. Mitochondria represent the major source of ROS, in addition, they are also the major ROS attack target, further aggravating OS [58]. In turn, OS can destroy mitochondria and release excessive ROS [59]. Such increasing ROS level can destroy ΔΨm through generating the permeability transition [60]. The increased mitochondrial ROS along with decreased ΔΨm levels predict mitochondrial impairments [61]. OS will damage mitochondrial ATP generation [62]. ATP is an essential energy molecule, which has a critical effect on different physiological events. Thus, the decreased ΔΨm is related to reduced ATP production, and this indicates mitochondrial dysfunction [62]. The increasing ROS generation within mitochondria and the decreased mitochondrial ΔΨm will induce mitochondrial DNA (mtDNA) injury and impair the electron-transport chain complex function [63]. The mtDNA mutations impair the electron-transport chain complexes within the oxidation respiratory chain, thus decreasing energy generation [64]. Mitophagy is identified as the key pathway for removing impaired mitochondria before cell death induction [65, 66]. Mitochondrial injury may cause PINK1 accumulation onto the outer mitochondrial membrane, and it has an important effect on Parkin recruitment [67]. Parkin contributes to mitochondrial protein ubiquitination and subsequently induces mitophagy [68]. In our study, BPA exposure in pregnant ewes increased ROS content, decreased ATP generation, complexes I–IV activities, and ΔΨm in placental [7] and ileal mitochondria, and activated mitophagy (PINK1, Parkin, ULK1, Beclin-1, and LC3-II/LC3-I) relative to CON pregnant ewes, indicating that BPA aggravated OS while leading to mitochondrial dysfunction within ileum and placenta of pregnant ewes. Microbial transplantation is a reliable method to analyze the involvement of gut microbiota in the defense mechanism of hosts with close genetic backgrounds [69]. According to our results, antibiotics-treated mice were orally administered with gut microbiota from pregnant ewes of CON and BPA groups, and GMT nearly exerted identical effects on ROS production, ΔΨm, ATP contents, mitophagy and activities of chondriosome’s complexes within placenta and ileum of mice. Therefore, gut microflora contributes to placental and ileal chondriosome function in the BPA-treated pregnant ewes.

Mitochondrial dysfunction has been related to elevated ROS production via organelles; alternatively, it may send signals onto the endoplasmic reticulum surface to increase ROS production [31]. Additionally, ROS induces ERS together with systemic inflammation features of metabolic syndrome [70]. Modulation of ERS can be important for the survival-mortality balance, which can be achieved by regulating apoptosis and autophagy upon diverse stresses [71]. In our study, BPA was found to initiate ERS within the placenta and ileum in pregnant ewes and activate unfolded protein response (UPR). Typically, UPR may increase ERS marker levels (GRP78, CHOP10, ATF4, ATF6) in vitro and in vivo, finally causing apoptosis featured by cytological changes [72]. Apoptosis within the placenta may induce poor pregnancy outcomes, like deformity, stillbirth, FGR, or premature birth [73]. According to our results, the elevated pro-apoptotic gene levels, such as Bax, caspase-9, caspase-8, and caspase-3, along with the reduced anti-apoptotic gene Bcl-2 expression suggested that both intrinsic and extrinsic apoptotic pathways were related to placentae [7] and the ileum of BPA-exposed pregnant ewe models. Autophagy and ERS are cytoprotective mechanisms that are triggered in the nonphysiological situation [74]. However, extended autophagy because of ERS activation can lead to cell death [75]. BPA treatment markedly increased the autophagy-related gene and protein levels (Parkin, PINK1, ULK1, Beclin-1, and LC3) together with LC3II/3I ratio within the ewe placenta of our previous study [7] and ileum in this study. Collectively, BPA-mediated FGR through activation of placental apoptosis based on increasing autophagy and ERS; moreover, BPA caused placental autophagy by stimulating ERS. Additionally, in our present study, compared with GMT (CON) mice, GMT (BPA) mice had a significant reduction in fetal weight with an increase in ERS, apoptosis, and autophagy of the placenta and ileum compared to the GMT (CON) mice. Consequently, BPA-induced apoptosis and FGR by activating autophagy and ERS within the placenta and ileum through modulating gut microbiota in our pregnant ewe model.

Gut microbiota is found to have a critical effect on metabolic processing, cell homeostasis and energy generation [76]. Gut dysbiosis may be related to placental insufficiency, a major factor causing FGR [77]. As discovered by integrating microbiomes with metabolite profiles of human cohorts, FGR patients may experience gut dysbiosis as well as metabolic disorders, thus facilitating disease pathogenesis [78]. According to our results, obvious alterations in gut microbiota could be seen in BPA-mediated FGR. Consequently, gut dysbiosis might induce FGR with the assistance of GMT. The ‘gut–placenta’ axis, which could be important for clarifying FGR etiology, was put forward. Further understanding of gut microbiome and placenta function can help to understand the relation of gut microbiome with pregnancy outcome.

Gut microbiota facilitates host physiology by producing various metabolites, like LPS and VFAs [79]. In this work, colonic LPS level was elevated and VFA contents (acetate, isobutyrate, butyrate, and propionate) were decreased after BPA application, which might be associated with alterations of gut microbial structure. Gut microbiota, together with the gut metabolites, is tightly associated with placental and intestinal functions [80, 81]. For example, intestinal-derived endotoxin can trigger inflammatory responses within the maternal placenta [76]. Maternal intestinal VFAs can enhance placental integrity while promoting fetal growth and placental vascularization [82–84]. The higher VFAs contents in the intestine will affect epithelial intactness as well as additional regional gut mucosal physiology [85]. Butyrate, a prominent VFA, is produced by gut microbiota via stodgy carbohydrate fermentation [86]. It can facilitate the growth and differentiation of intestinal epithelial cells and modulate intestinal antioxidant activities [87]. Clostridium is a critical genus of butyrate-generating bacteria, which has an essential effect on maintaining gut homeostasis [88]. Most bacteria belonging to Clostridium and Lactobacillus genera can be beneficial and have important effects on preventing gut pathogens while maintaining host homeostasis and immunity [89]. Veillonella accounts for the main opportunistic pathogens related to chronic inflammation and unfavorable pregnancy outcomes [90]. As reported previously, maternal gut microbiota is important in gestation, moreover, Bifidobacterium regulates placental structure, and maternal physiology, together with nutrient transporter ability, and affects fetal growth and glycemia [91]. The ratio of Firmicutes/Bacteroidetes is a possible biomarker of metabolic syndrome such as obesity and related dysfunctions, and there is an increase in Firmicutes, which has been associated with an increase in the need for energy storage [92]. According to our results, Bifidobacterium, Lactobacillus, and butyrate-producing Clostridium abundances decreased, but Veillonella abundance and Firmicutes/Bacteroidetes ratio increased within the colon of the BPA-treated pregnant ewes. Therefore, gut microbiota together with their associated metabolites could be the new targets for the treatment of BPA-mediated placental impairment. To further examine the relation of BPA-induced maternal microbial changes with the injury of the placenta and intestine, we transplanted microbiota from CON and BPA groups of pregnant ewes into microbiota-free mice. Results from GMT (CON) and GMT (BPA) mice suggested the close impacts on colonic microbial composition, VFAs, LPS levels, intestinal and placental apoptosis and OS in ewes from CON and BPA groups. Therefore, our results suggested the causal function of gut microbiota in the apoptosis, OS, ERS, autophagy, and mitochondrial function of maternal intestines and placenta in pregnant ewe models.

## Conclusion

This work illustrated the mechanism underlying the role of the gut microbiota and gut-placental axis in the BPA-triggered FGR and placental OS, apoptosis, autophagy, and ERS. The BPA changed the diversity and composition of maternal colonic microbiota, thus decreasing VFAs contents while increasing LPS content, which penetrated the blood-placenta via placental and gut functional defects. Drugs and probiotics functioning via the gut-placental axis through modulating the balance of gut microbiota can be the novel candidate direction to alleviate the gut-derived placental impairment or FGR. Moreover, the BPA administration in the sheep could trigger some unspecific immune/inflammatory response with very species-particular molecules (for example IgA antibodies) that could lead to an immune/inflammatory response in mice. This response, although triggered by BPA and included in the transplant, may not be exclusively related to microbiota dysbiosis, and further research is needed to elucidate it.

### Supplementary Information

**Additional file 1: Table S1.** Ingredient and nutrient composition of the experimental diets on a dry matter basis. **Table S2.** Primer sequences used in the real-time PCR. **Table S3.** Details of antibodies used for western blotting. **Fig. S1.** Representative histologic alteration of the ileal morphology in sheep from the (A) CON and (B) BPA groups, and ileal morphology in mice from the (C) GMT (CON) and (D) GMT (BPA) groups. All sections were stained with hematoxylin and eosin and examined at 100× magnification. Scale bar = 100 µm.

## Data Availability

All data relevant to the study are included in the article or uploaded as supplementary information. Data are available on reasonable request. Data generated and analyzed during this study are available from the corresponding author upon reasonable request.

## Abbreviations

ACE: Abundance-based coverage estimator
ATP: Adenosine triphosphate
ATF: Activating transcription factor
BPA: Bisphenol A
CHOP10: C/EBP homologous protein 10
CAT: Catalase
ERS: Endoplasmic reticulum stress
FGR: Fetal growth restriction
GRP78: Glucose-regulated protein 78
GPX1: Glutathione peroxidase 1
GSH-Px: Glutathione peroxidase
GMT: Gut microbiota transplantation
HO-1: Heme oxygenase-1
LC3: Microtubule-associated protein light chain 3
LPS: Lipopolysaccharide
MDA: Malonaldehyde
Nrf2: Nuclear factor erythroid 2-related factor 2
NQO1: Quinone oxidoreductase 1
OS: Oxidative stress
PINK1: PTEN-induced putative kinase 1
ROS: Reactive oxygen species
SOD: Superoxide dismutase
T-AOC: Total antioxidant capacity
ULK1: Unc-51 like autophagy activating kinase 1
VCR: Villous height/crypt depth ratio
VFAs: Volatile fatty acids
