# Supplementary material for: Gut microbiota contributes to bisphenol A-induced maternal intestinal and placental apoptosis, oxidative stress, and fetal growth restriction in pregnant ewe model by regulating gut-placental axis
Source: Microbiome. 2024 Feb 17;12:28. doi: 10.1186/s40168-024-01749-5 (PMC10874076; doi:10.1186/s40168-024-01749-5)

**Table S 1** Ingredient and nutrient composition of the experimental diets on a dry matter basis.

| Items^1^ | Diet 1  (0 to 90 d of gestation) | Diet 2  (91 to110 d of gestation) |
| --- | --- | --- |
| Ingredient, % |  |  |
| Chinese wild rye | 50.00 | 45.00 |
| Corn | 35.12 | 31.32 |
| Soybean meal | 12.00 | 20.00 |
| Dicalcium phosphate | 1.67 | 2.34 |
| Calcium carbonate | 0.41 | 0.54 |
| Salt | 0.50 | 0.50 |
| Mineral/vitamin premix^2^ | 0.30 | 0.30 |
| Total | 100 | 100 |
| Nutrient composition (analysed)^3^ |  |  |
| GE, MJ/kg | 17.63 | 18.49 |
| ME, MJ/kg | 9.23 | 10.03 |
| CP, % | 9.98 | 13.59 |
| MP, % | 6.42 | 8.69 |
| EE, % | 4.21 | 4.59 |
| NDF, % | 37.12 | 32.57 |
| ADF, % | 20.98 | 18.93 |
| Ca, % | 0.57 | 0.81 |
| P, % | 0.45 | 0.69 |

^1^ADF, acid detergent fibre; CP, crude protein; EE, ether extract; GE, gross energy; ME, metabolizable energy; MP, Metabolizable protein; NDF, neutral detergent fibre.

^2^The premix provided the following nutrients per kilogram of diet: 30,000 IU vitamin A, 10,000 IU vitamin D, 100 mg vitamin E, 90 mg Fe, 12.5 mg Cu, 50 mg Mn, 100 mg Zn, 0.3 mg Se, 0.8 mg I and 0.5 mg Co.

^3^Nutrient levels are analysed values. Energy and protein values were estimated according to NRC (2007). Methods for chemical analysis of the diet have been reported in a previous study (Zhang et al., 2021a).

**Table S2** Primer sequences used in the real-time PCR.

| Gene | Sequence (5’–3’) | GenBank accession number |
| --- | --- | --- |
| **Sheep**  Pro-apoptotic and anti-apoptotic genes  *Fas* | F: TTTTGCTGTCAGCCTTGTCC  R: TGTTCCACTTCTAGCCCATG | NM_001123003.1 |
| *Bax* | F：ATGGGCTGGACATTGGACTT  R：ACTGTCTGCCATGTGGGTGT | AF163774.1 |
| *Bcl-2* | F：CGAGTGGCGGCTGAAAT  R：GGTCTGCCATGTGGGTGTC | HM630309.1 |
| *P53* | F：TTCCCCTTCCCTCAACAAGC  R：GCGCGTAAATTCCCTTCCAC | NM_001009403.1 |
| *Caspase 3* | F：TCAGGGAAACCTTCACGAGC  R：CCTCGGCAGGCCTGAATAAT | XM_027962551.1 |
| *Caspase 8* | F：TGAAGGTTCCAGGATTCGCC  R：GGCTTAGGAACTTGAGGGCA | XM_004004822.1 |
| *Caspase 9* | F： GCCAAGCCAAGGAAAACTCG  R： CACGGCAGAAGTTCACGTTG | XM_042257438.1 |
| Antioxidant-related genes |  |  |
| *CAT* | F：CCATCTGAAAGACGCACAGC | GQ421282.1 |
|  | r：ATGCGGGAGCCATACTCAG |  |
| *GPx1* | F：GCAACCAGTTTGGGCATCAG | JF728302.1 |
|  | r：GCCATTCACCTCGCACTTTT |  |
| *SOD2* | F: TCACAGCATCTTCTGGACAA | NM_001280703 |
|  | R: TGCTCCTTATTGAAGCCAAG |  |
| *Nrf2*  *HO-1*  *NQO1* | F: CGAGCCGGTGTGAGTAGA  R: TTCCGTGGCCCAGTGTAAAG  F: TGGAGGAGGAGATAGAACGC  R: CCTGGAGTCGCTGAACATAG  F: TTCTGTGGCTTCCAGGTCTT  R: TCCAGACGTTTCTTCCATCC | AY369137.1  XM_015094843.1  XM_004015102.3 |
| ER stress-related genes  *CHOP10*  *GRP78*  *ATF4*  *ATF6*  Autophagy-related genes | F: AGGACCACCAGAGGTCACAC  R: TGCCACTTTCCTTTCGTTTT  F: TGAAACTGTGGGAGGTGTCA  R: TCGAAAGTTCCCAGAAGGTG  F: CGAGGTGTTTGTGGGGGACT  R: AGGAGCCTGCCTTAGCCTTG  F: AACCAGTCCTTGCTGTTGCT  R: CTTCTTCTTGCGGGACTGAC | AY943948.1  DQ029323.1  GAAI01000637.1  AY942654 |
| *ULK1* | F: CAACCACAAAGACCGCATGG  R: CCCTGAGCTCGGGTATGAG | XM_015101564.1 |
| *Beclin-1* | F: CGGACCGGAAAGTAGCTGAAG  r: GCTGTGGCAAGTAATGGAGC | XM_004012945.3 |
| *LC3* | f: TGTCAACATGAGCGAGTTGGT  r: GCTCGTAGATGTCCGCGATG | XM_012114930.2 |
| *β-actin* | F: GCTCTTCCAGCCGTCCTT  R: TGAAGGTGGTCTCGTGAATGC | NM_001009784.1 |
| **Mice**  Pro-apoptotic and anti-apoptotic genes  *Fas* | F: ACTGCGATTCTTCTGGCTGT  R: GCGATTTCTGGGACTTTGTT | DQ846749.1 |
| *Bax* | F: TGGAGATGAACTGGACAGCAATAT  R: GCAAAGTAGAAGAGGGCAACCAC | NM_007527. 3 |
| *Bcl-2* | F: CATTATCAATGATGTACCATG  R: GCAGTAAATAGCTGATTCGAC | NM_009741.3 |
| *P53* | F: TACAAGAAGTCACAGCACAT  R: GATAGGTCGGCGGTTCAT | AB020317.1 |
| *Caspase 3* | F: CAAAGCGCAGTGTCCTGCGG  R: ACCCCGGCAGGCCTGAATGA | NM_009810.3 |
| *Caspase 8* | F: GATGTTGGAGGAAGGCAATC  R: ATTCCAACTCGCTCACTTCT | AJ007749.1 |
| *Caspase 9* | F: TGACATCCTTGTGTCCTACTC  R: CCAGGAATCTGCTTGTAAGTC | AB019600.1 |
| Antioxidant-related genes |  |  |
| *CAT* | F: CCTCGTTCAGGATGTGGT TT  R: TCTGGTGATATCGTGGGTGA | NM_009804 |
| *GPx1* | F: CCTCAAGTACGTCCGACCTG  R: CAATGTCGTTGCGGCACACC | NM_001329528.1 |
| *SOD2* | F: GGCTGGCTTGGCTTCAATAA  R: GCGGAATAAGGCCTGTTGTT | NM_013671.3 |
| *Nrf2* | F: CGAGATATACGCAGGAGAGGTAAGA  R: GCTCGACAATGTTCTCCAGCTT | AH006764.2 |
| *HO-1* | F: CACGCCAGCCACACAGCACT  R: GTTGAGCAGGAAGGCGGTCT | NM_010442.2 |
| *NQO1* | F: ACAACGGTCCTTTCCAGAAT  R: TCCCAGACGGTTTCCAGACG | NM_008706.5 |
| ER stress-related genes |  |  |
| *CHOP10* | F: AGTGCATCTTCATACACCACCACA  R: CAGATCCTCATACCAGGCTTCCA | NM_007837.4 |
| *GRP78* | F: ATCGTGCCTCTCATTGGTGG  R: TAGTTGGAGGCCGCTGATTG | U16277.1 |
| *ATF4* | F: gggttctgtcttccactcca  R: aagcagcagagtcaggctttc | NM_009716.3 |
| *ATF6* | F: ATGGGTTCGGATATCGCTGT  R: TTCTTCTTCTTGCGCGACTG | NM_001081304.1 |
| Autophagy-related genes |  |  |
| *ULK1* | F: AGTTCTGGAGATTGCAGCCC  R: ACCACACTTTCCTGGAGCTG | NM_009469.3 |
| *Beclin-1* | F: GAAACTGGACACGAGCTTCAAGA  R: ACCATCCTGGCGAGTTTCAATA | NM_001034117.1 |
| *LC3* | F: CCTGGACAAGACCAAGTTCC  R: GTCTCCTGCGAGGCATAAAC | NM_026160.4 |
| *β-actin* | F: AGCCATGTACGTAGCCATCC  R: CTCTCAGCTGTGGTGGTGAA | NM007393 |

*ATF6*, activating transcription factor 6; *ATF4*, activating transcription factor 4; *Bax*, Bcl-2-associated X protein; *Bcl-2*, B-cell lymphoma/leukaemia 2; *CAT*, catalase; *CHOP10*, C/EBP homologous protein 10; ER, endoplamic reticulum; F, forward primer; *GRP78*, glucose-regulated protein 78; *GPx1*, glutathione peroxidase 1; *HO-1*, heme oxygenase-1; *LC3*, microtubule associated protein light chain 3; *Nrf2*, nuclear factor erythroid 2-related factor 2; *NQO1*, quinone oxidoreductase 1; R, reverser primer; *SOD2*, superoxide dismutase 2; *ULK1*, unc-51 like autophagy activating kinase 1.

**Table S3** Details of antibodies used for western blotting.

| Antibodies | Cat NO. | Source | Dilutions of Western blot |
| --- | --- | --- | --- |
| Anti-GPx1 | 29329-1-AP | Proteintech (CHI, USA) | 1:2000 |
| Anti-CAT | 21260-1-AP | Proteintech (CHI, USA) | 1:2000 |
| Anti-SOD2 | 24127-1-AP | Proteintech (CHI, USA) | 1:5000 |
| Anti-Caspase 3 | 19677-1-AP | Proteintech (CHI, USA) | 1:1000 |
| Anti-Bcl-2 | 26593-1-AP | Proteintech (CHI, USA) | 1:1000 |
| Anti-Bax | 50599-2-Ig | Proteintech (CHI, USA) | 1:3000 |
| Anti-Fas  Anti-P53 | 13098-1-AP  10442-1-AP | Proteintech (CHI, USA)  Protein Tech (CHI, USA) | 1:1000  1:5000 |
| Anti-GRP78 | 11587-1-AP | Protein Tech (CHI, USA) | 1:2000 |
| Anti-CHOP10 | 15204-1-AP | Protein Tech (CHI, USA) | 1:1000 |
| Anti-ATF6 | 24169-1-AP | Protein Tech (CHI, USA) | 1:2000 |
| Anti-Parkin | 14060-1-AP | Protein Tech (CHI, USA) | 1:1500 |
| Anti-PINK1 | 23274-1-AP | Protein Tech (CHI, USA) | 1:1000 |
| Anti-LC3 | 14600-1-AP | Protein Tech (CHI, USA) | 1:2000 |
| Anti-Beclin1 | 11306-1-AP | Protein Tech (CHI, USA) | 1:2000 |
| Anti-β-actin | 20536-1-AP | Protein Tech (CHI, USA) | 1:1500 |
| Anti-VDAC | 10866-1-AP | Protein Tech (CHI, USA) | 1:1000 |
| HRP-labelled goat anti-rabbit IgG | A0208 | Beyotime Biotech (Shanghai, China) | 1:1000 |

ATF6, activating transcription factor 6; Bax, Bcl-2-associated X protein; Bcl-2, B-cell lymphoma/leukaemia 2; CAT, catalase; CHOP10, C/EBP homologous protein 10; GPx1, glutathione peroxidase 1; GRP78, glucose-regulated protein 78; HRP, horseradish peroxidase; LC3, microtubuleassociated protein light chain 3; PINK1, PTEN induced putative kinase 1; SOD2, superoxide dismutase 2; VDAC, voltage dependent anion channel.

**Fig. S1** Representative histologic alteration of the ileal morphology in sheep from the (A) CON and (B) BPA groups, and ileal morphology in mice from the (C) GMT (CON) and (D) GMT (BPA) groups. All sections were stained with hematoxylin and eosin and examined at 100× magnification. Scale bar = 100 µm.

**A．CON B．BPA**


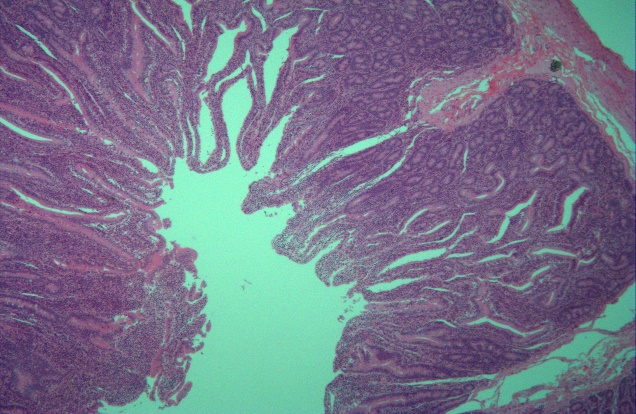

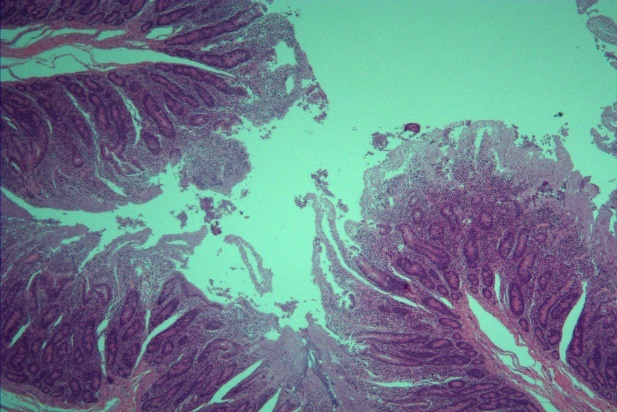


**C．GMT (CON) D．GMT (BPA)**


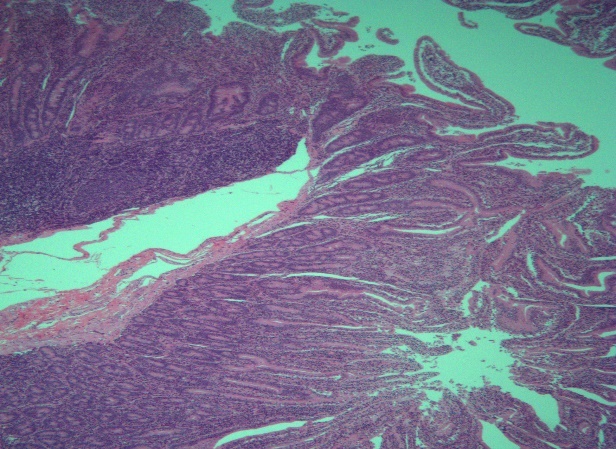

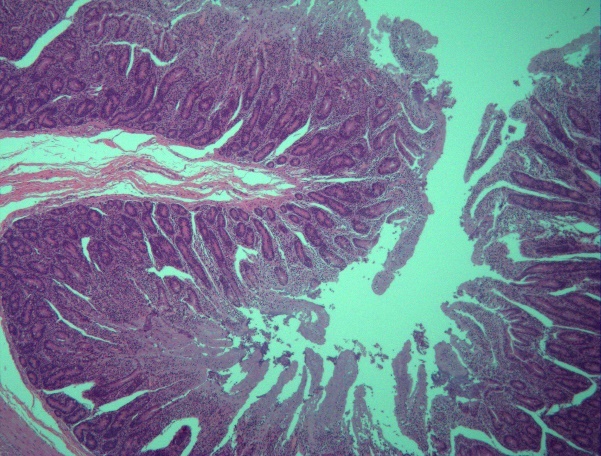

Supplement: Supplementary file 2 — Additional file 1: Table S1. Ingredient and nutrient composition of the experimental diets on a dry matter basis. Table S2. Primer sequences used in the real-time PCR. Table S3. Details of antibodies used for western blotting. Fig. S1. Representative histologic alteration of the ileal morphology in sheep from the (A) CON and (B) BPA groups, and ileal morphology in mice from the (C) GMT (CON) and (D) GMT (BPA) groups. All sections were stained with hematoxylin and eosin and examined at 100× magnification. Scale bar = 100 µm. [file 40168_2024_1749_MOESM1_ESM.docx]
